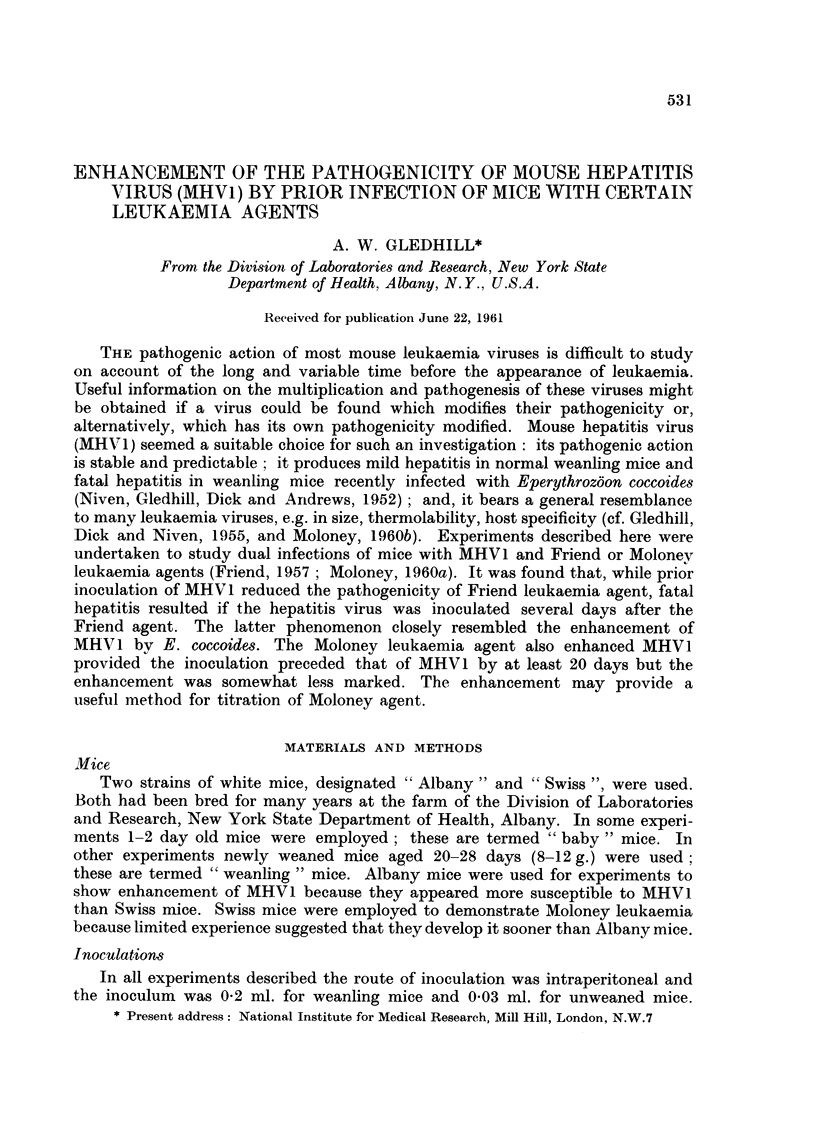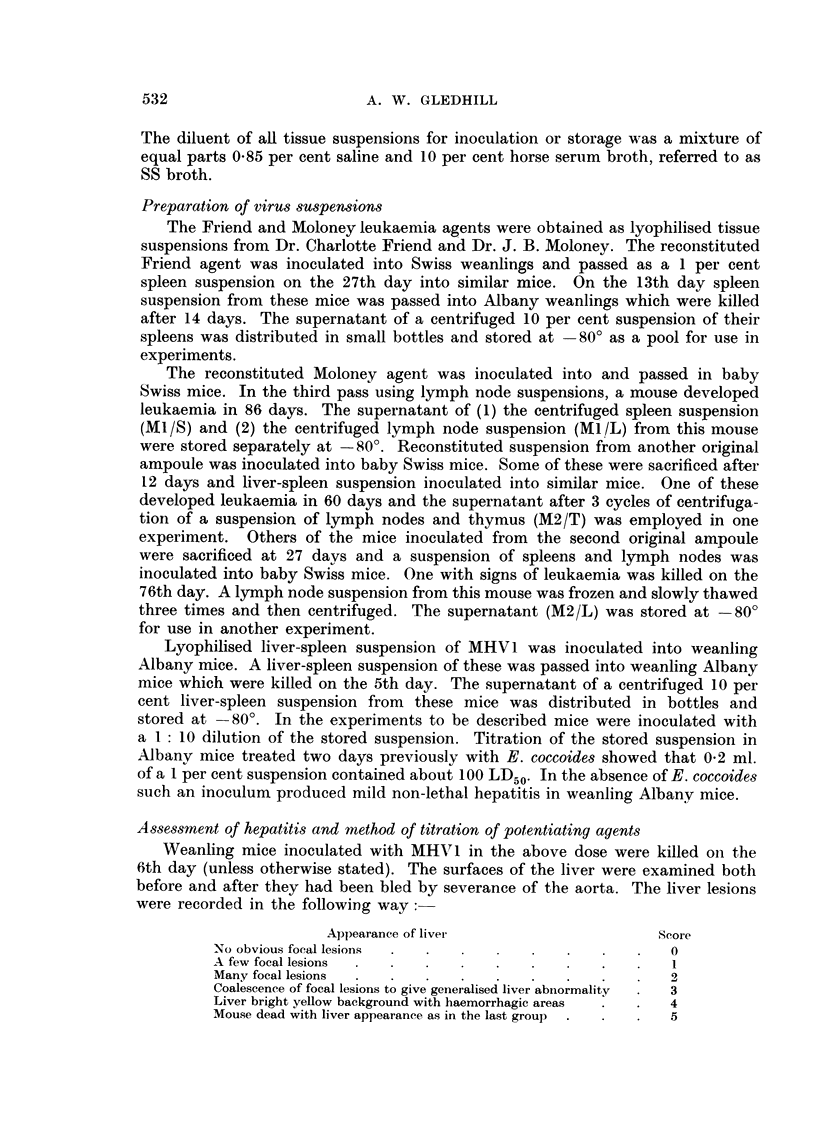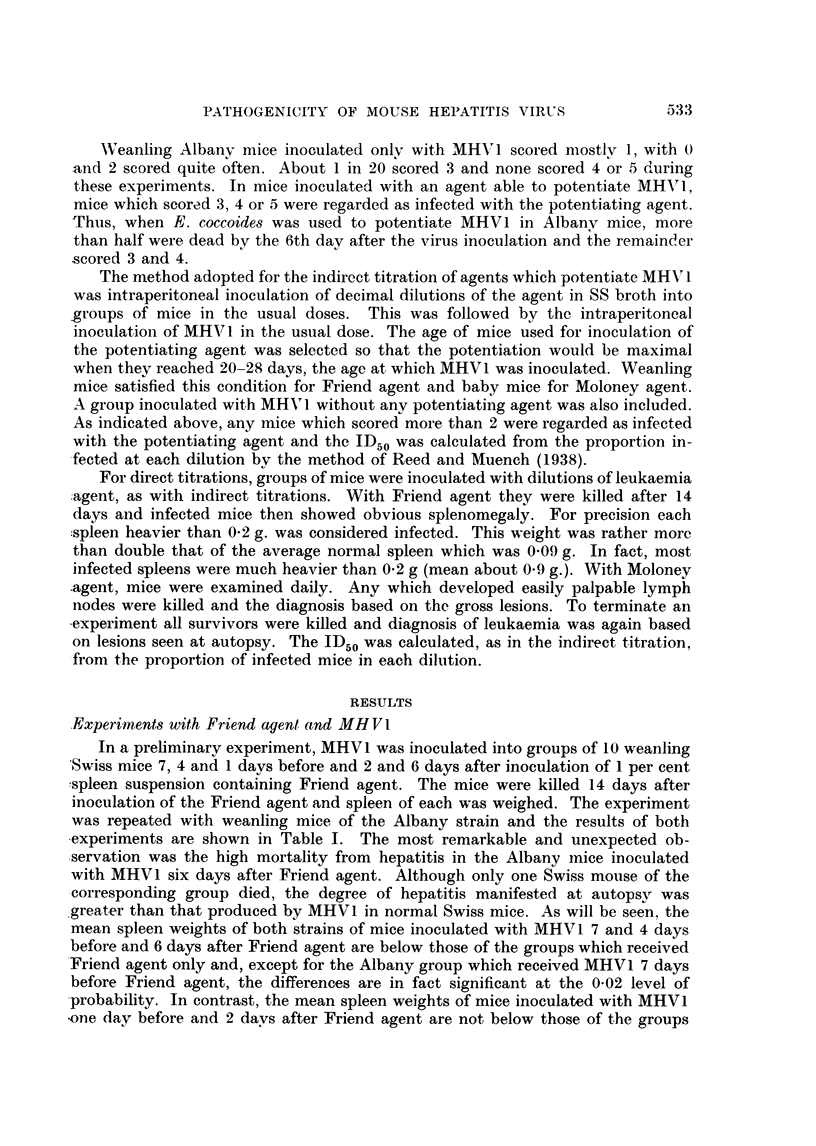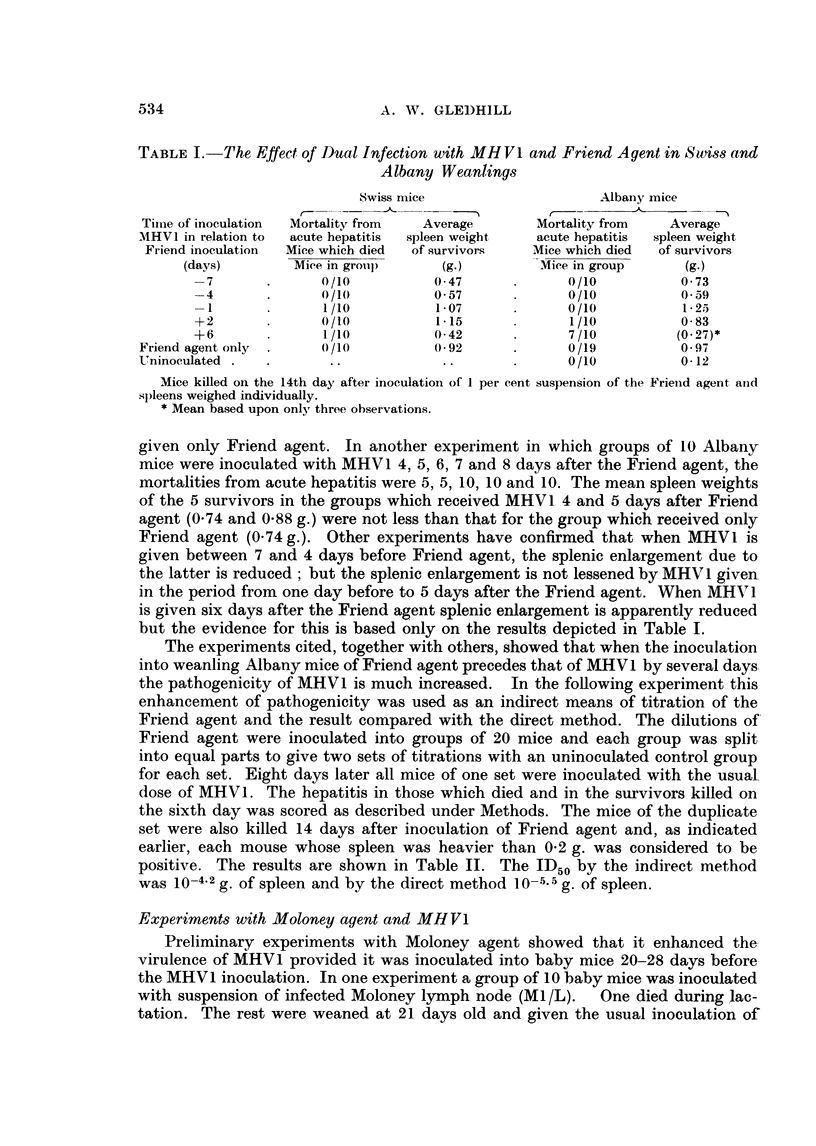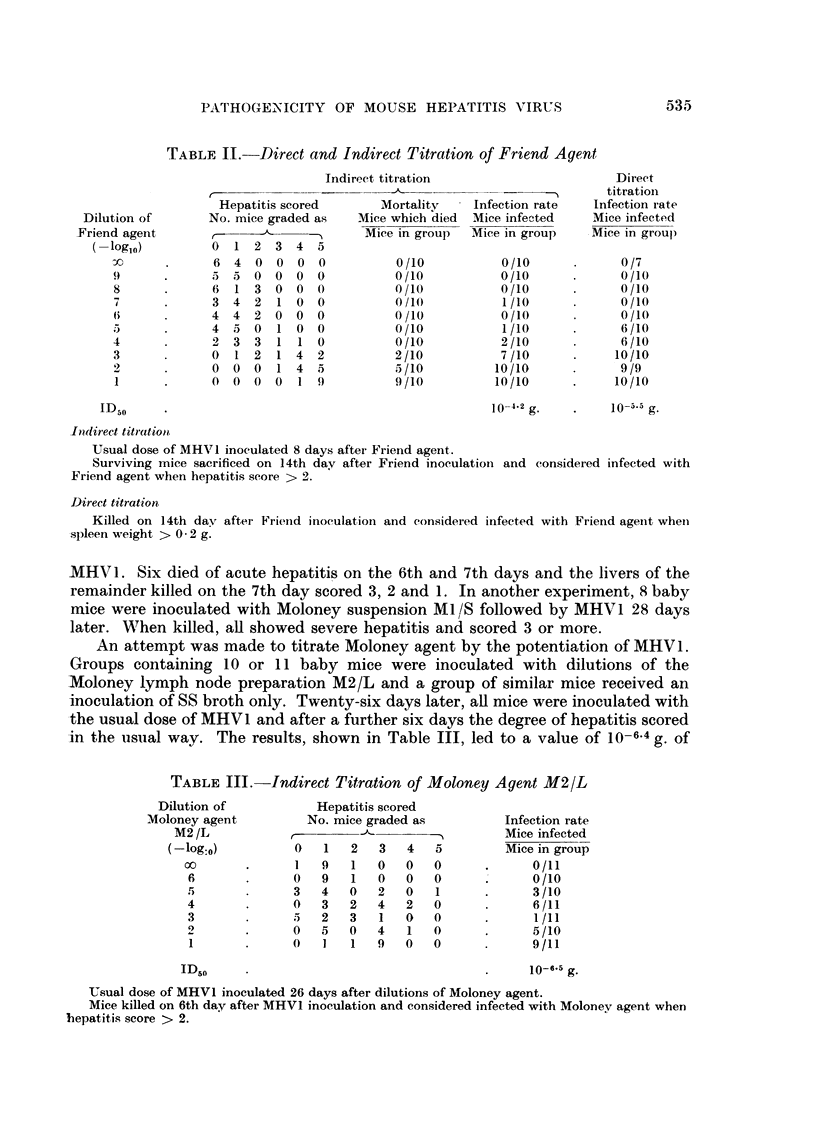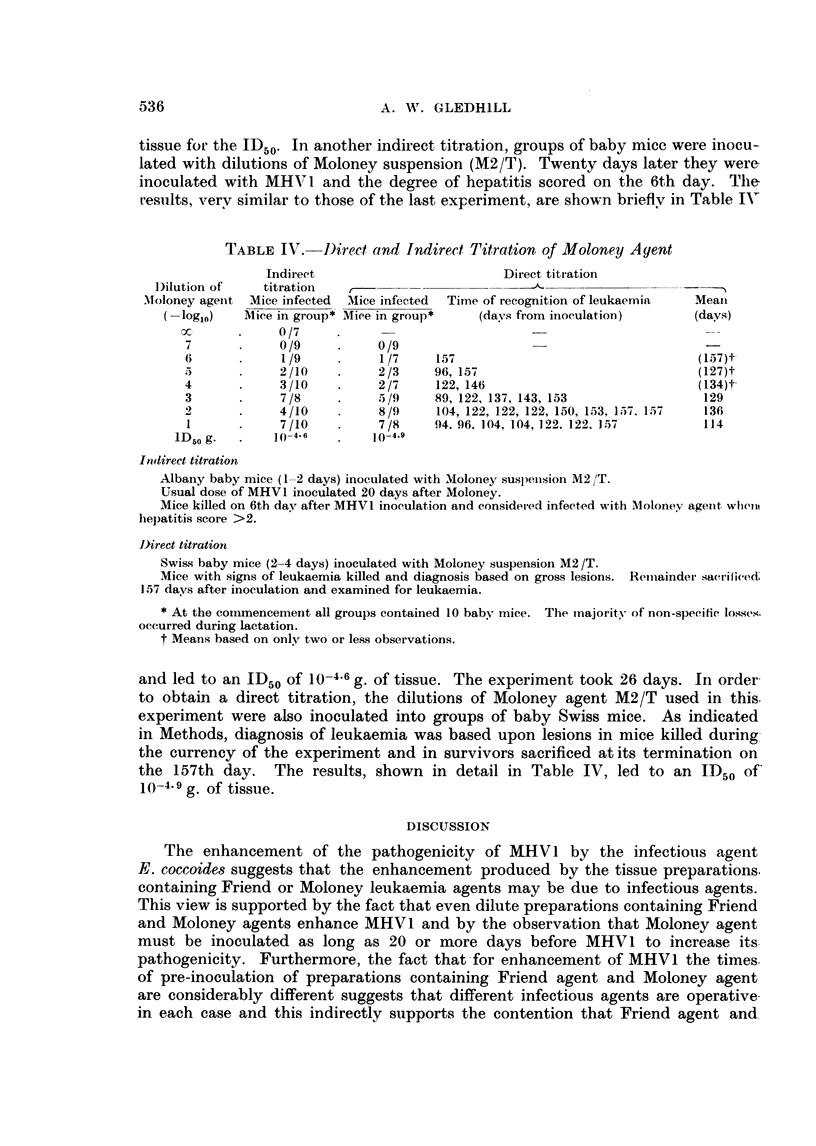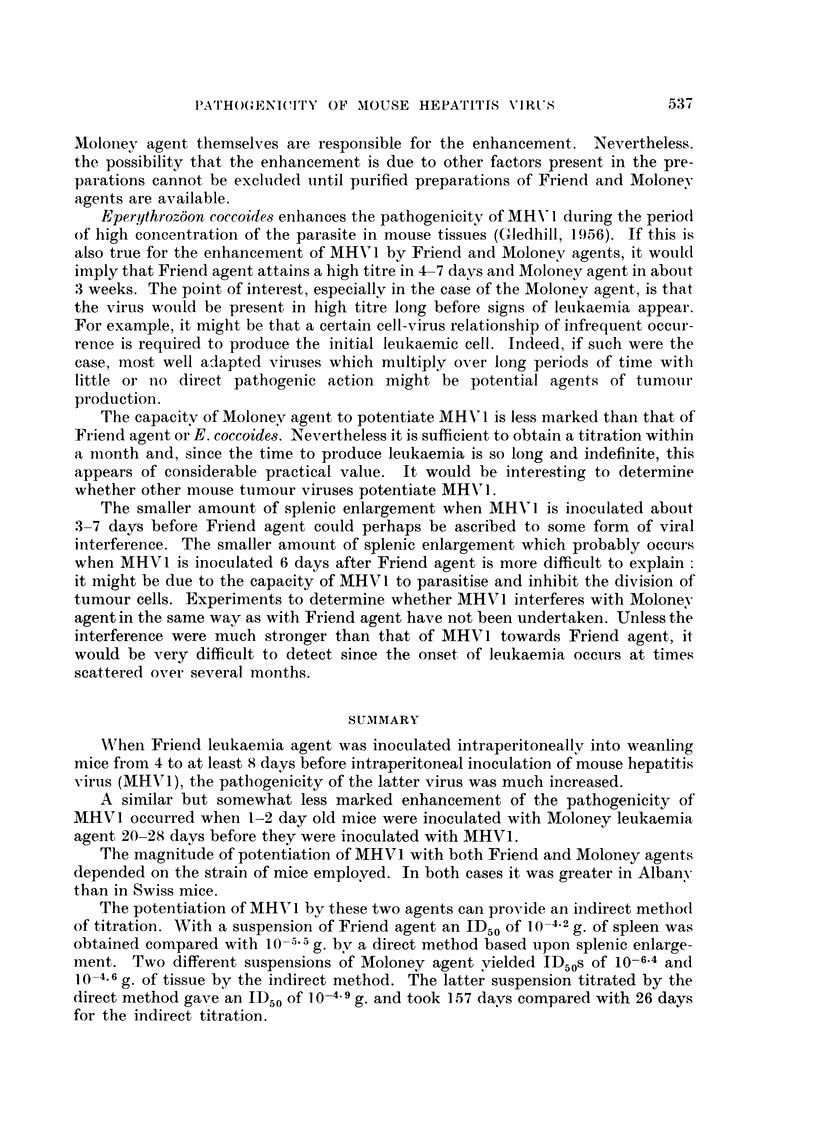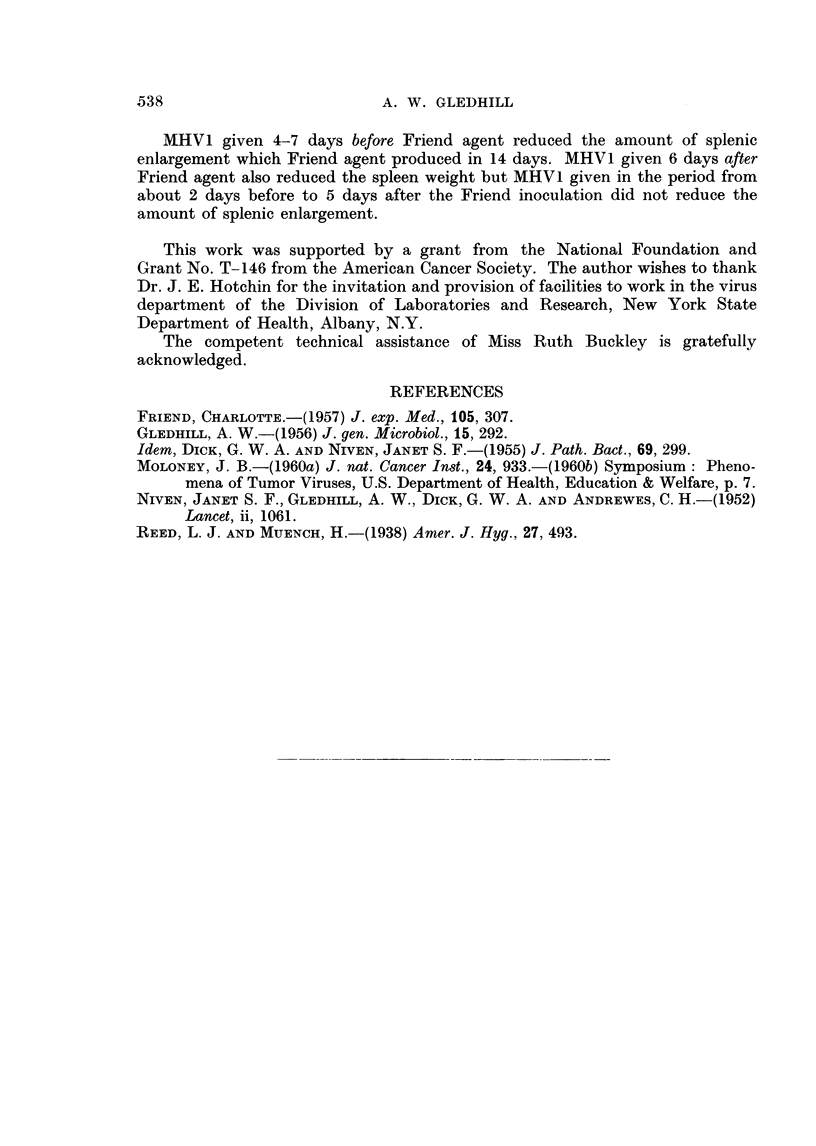# Enhancement of the Pathogenicity of Mouse Hepatitis Virus (MHV1) By Prior Infection of Mice with Certain Leukaemia Agents

**DOI:** 10.1038/bjc.1961.63

**Published:** 1961-09

**Authors:** A. W. Gledhill


### ENHANCEMENT OF THE PATHOGENICITY OF MOUSE HEPATITIS VIRUS (MHV1) BY PRIOR INFECTION OF MICE WITH CERTAIN LEUKAEMIA AGENTS

A. W. GLEDHILL*

*From the Division of Laboratories and Research, New York State Department of Health, Albany, N.Y., U.S.A.*

Received for publication June 22, 1961

THE pathogenic action of most mouse leukaemia viruses is difficult to study on account of the long and variable time before the appearance of leukaemia. Useful information on the multiplication and pathogenesis of these viruses might be obtained if a virus could be found which modifies their pathogenicity or, alternatively, which has its own pathogenicity modified. Mouse hepatitis virus (MHV1) seemed a suitable choice for such an investigation : its pathogenic action is stable and predictable ; it produces mild hepatitis in normal weanling mice and fatal hepatitis in weanling mice recently infected with *Eperythrozöon coccoides* (Niven, Gledhill, Dick and Andrews, 1952) ; and, it bears a general resemblance to many leukaemia viruses, e.g. in size, thermolability, host specificity (cf. Gledhill, Dick and Niven, 1955, and Moloney, 1960*b*). Experiments described here were undertaken to study dual infections of mice with MHV1 and Friend or Moloney leukaemia agents (Friend, 1957 ; Moloney, 1960*a*). It was found that, while prior inoculation of MHV1 reduced the pathogenicity of Friend leukaemia agent, fatal hepatitis resulted if the hepatitis virus was inoculated several days after the Friend agent. The latter phenomenon closely resembled the enhancement of MHV1 by *E. coccoides*. The Moloney leukaemia agent also enhanced MHV1 provided the inoculation preceded that of MHV1 by at least 20 days but the enhancement was somewhat less marked. The enhancement may provide a useful method for titration of Moloney agent.

### MATERIALS AND METHODS

### *Mice*

Two strains of white mice, designated " Albany " and " Swiss ", were used. Both had been bred for many years at the farm of the Division of Laboratories and Research, New York State Department of Health, Albany. In some experiments 1–2 day old mice were employed ; these are termed " baby " mice. In other experiments newly weaned mice aged 20–28 days (8–12 g.) were used ; these are termed " weanling " mice. Albany mice were used for experiments to show enhancement of MHV1 because they appeared more susceptible to MHV1 than Swiss mice. Swiss mice were employed to demonstrate Moloney leukaemia because limited experience suggested that they develop it sooner than Albany mice.

### *Inoculations*

In all experiments described the route of inoculation was intraperitoneal and the inoculum was 0·2 ml. for weanling mice and 0·03 ml. for unweaned mice.

* Present address : National Institute for Medical Research, Mill Hill, London, N.W.7

The diluent of all tissue suspensions for inoculation or storage was a mixture of equal parts 0·85 per cent saline and 10 per cent horse serum broth, referred to as SS broth.

*Preparation of virus suspensions*

The Friend and Moloney leukaemia agents were obtained as lyophilised tissue suspensions from Dr. Charlotte Friend and Dr. J. B. Moloney. The reconstituted Friend agent was inoculated into Swiss weanlings and passed as a 1 per cent spleen suspension on the 27th day into similar mice. On the 13th day spleen suspension from these mice was passed into Albany weanlings which were killed after 14 days. The supernatant of a centrifuged 10 per cent suspension of their spleens was distributed in small bottles and stored at $-80°$ as a pool for use in experiments.

The reconstituted Moloney agent was inoculated into and passed in baby Swiss mice. In the third pass using lymph node suspensions, a mouse developed leukaemia in 86 days. The supernatant of (1) the centrifuged spleen suspension (M1/S) and (2) the centrifuged lymph node suspension (M1/L) from this mouse were stored separately at $-80°$. Reconstituted suspension from another original ampoule was inoculated into baby Swiss mice. Some of these were sacrificed after 12 days and liver-spleen suspension inoculated into similar mice. One of these developed leukaemia in 60 days and the supernatant after 3 cycles of centrifugation of a suspension of lymph nodes and thymus (M2/T) was employed in one experiment. Others of the mice inoculated from the second original ampoule were sacrificed at 27 days and a suspension of spleens and lymph nodes was inoculated into baby Swiss mice. One with signs of leukaemia was killed on the 76th day. A lymph node suspension from this mouse was frozen and slowly thawed three times and then centrifuged. The supernatant (M2/L) was stored at $-80°$ for use in another experiment.

Lyophilised liver-spleen suspension of MHV1 was inoculated into weanling Albany mice. A liver-spleen suspension of these was passed into weanling Albany mice which were killed on the 5th day. The supernatant of a centrifuged 10 per cent liver-spleen suspension from these mice was distributed in bottles and stored at $-80°$. In the experiments to be described mice were inoculated with a 1 : 10 dilution of the stored suspension. Titration of the stored suspension in Albany mice treated two days previously with *E. coccoides* showed that 0·2 ml. of a 1 per cent suspension contained about 100 $LD_{50}$. In the absence of *E. coccoides* such an inoculum produced mild non-lethal hepatitis in weanling Albany mice.

*Assessment of hepatitis and method of titration of potentiating agents*

Weanling mice inoculated with MHV1 in the above dose were killed on the 6th day (unless otherwise stated). The surfaces of the liver were examined both before and after they had been bled by severance of the aorta. The liver lesions were recorded in the following way :—

| Appearance of liver | Score |
|---|---|
| No obvious focal lesions | 0 |
| A few focal lesions | 1 |
| Many focal lesions | 2 |
| Coalescence of focal lesions to give generalised liver abnormality | 3 |
| Liver bright yellow background with haemorrhagic areas | 4 |
| Mouse dead with liver appearance as in the last group | 5 |

Weanling Albany mice inoculated only with MHV1 scored mostly 1, with 0 and 2 scored quite often. About 1 in 20 scored 3 and none scored 4 or 5 during these experiments. In mice inoculated with an agent able to potentiate MHV1, mice which scored 3, 4 or 5 were regarded as infected with the potentiating agent. Thus, when *E. coccoides* was used to potentiate MHV1 in Albany mice, more than half were dead by the 6th day after the virus inoculation and the remainder scored 3 and 4.

The method adopted for the indirect titration of agents which potentiate MHV1 was intraperitoneal inoculation of decimal dilutions of the agent in SS broth into groups of mice in the usual doses. This was followed by the intraperitoneal inoculation of MHV1 in the usual dose. The age of mice used for inoculation of the potentiating agent was selected so that the potentiation would be maximal when they reached 20–28 days, the age at which MHV1 was inoculated. Weanling mice satisfied this condition for Friend agent and baby mice for Moloney agent. A group inoculated with MHV1 without any potentiating agent was also included. As indicated above, any mice which scored more than 2 were regarded as infected with the potentiating agent and the $ID_{50}$ was calculated from the proportion infected at each dilution by the method of Reed and Muench (1938).

For direct titrations, groups of mice were inoculated with dilutions of leukaemia agent, as with indirect titrations. With Friend agent they were killed after 14 days and infected mice then showed obvious splenomegaly. For precision each spleen heavier than 0·2 g. was considered infected. This weight was rather more than double that of the average normal spleen which was 0·09 g. In fact, most infected spleens were much heavier than 0·2 g (mean about 0·9 g.). With Moloney agent, mice were examined daily. Any which developed easily palpable lymph nodes were killed and the diagnosis based on the gross lesions. To terminate an experiment all survivors were killed and diagnosis of leukaemia was again based on lesions seen at autopsy. The $ID_{50}$ was calculated, as in the indirect titration, from the proportion of infected mice in each dilution.

### RESULTS

### *Experiments with Friend agent and MHV1*

In a preliminary experiment, MHV1 was inoculated into groups of 10 weanling Swiss mice 7, 4 and 1 days before and 2 and 6 days after inoculation of 1 per cent spleen suspension containing Friend agent. The mice were killed 14 days after inoculation of the Friend agent and spleen of each was weighed. The experiment was repeated with weanling mice of the Albany strain and the results of both experiments are shown in Table I. The most remarkable and unexpected observation was the high mortality from hepatitis in the Albany mice inoculated with MHV1 six days after Friend agent. Although only one Swiss mouse of the corresponding group died, the degree of hepatitis manifested at autopsy was greater than that produced by MHV1 in normal Swiss mice. As will be seen, the mean spleen weights of both strains of mice inoculated with MHV1 7 and 4 days before and 6 days after Friend agent are below those of the groups which received Friend agent only and, except for the Albany group which received MHV1 7 days before Friend agent, the differences are in fact significant at the 0·02 level of probability. In contrast, the mean spleen weights of mice inoculated with MHV1 one day before and 2 days after Friend agent are not below those of the groups

TABLE I.—*The Effect of Dual Infection with MHV1 and Friend Agent in Swiss and Albany Weanlings*

| Time of inoculation MHV1 in relation to Friend inoculation (days) | Swiss mice: Mortality from acute hepatitis Mice which died / Mice in group | Swiss mice: Average spleen weight of survivors (g.) | Albany mice: Mortality from acute hepatitis Mice which died / Mice in group | Albany mice: Average spleen weight of survivors (g.) |
|---|---|---|---|---|
| −7 | 0/10 | 0·47 | 0/10 | 0·73 |
| −4 | 0/10 | 0·57 | 0/10 | 0·59 |
| −1 | 1/10 | 1·07 | 0/10 | 1·25 |
| +2 | 0/10 | 1·15 | 1/10 | 0·83 |
| +6 | 1/10 | 0·42 | 7/10 | (0·27)* |
| Friend agent only | 0/10 | 0·92 | 0/19 | 0·97 |
| Uninoculated | .. | .. | 0/10 | 0·12 |

Mice killed on the 14th day after inoculation of 1 per cent suspension of the Friend agent and spleens weighed individually.
* Mean based upon only three observations.

given only Friend agent. In another experiment in which groups of 10 Albany mice were inoculated with MHV1 4, 5, 6, 7 and 8 days after the Friend agent, the mortalities from acute hepatitis were 5, 5, 10, 10 and 10. The mean spleen weights of the 5 survivors in the groups which received MHV1 4 and 5 days after Friend agent (0·74 and 0·88 g.) were not less than that for the group which received only Friend agent (0·74 g.). Other experiments have confirmed that when MHV1 is given between 7 and 4 days before Friend agent, the splenic enlargement due to the latter is reduced ; but the splenic enlargement is not lessened by MHV1 given in the period from one day before to 5 days after the Friend agent. When MHV1 is given six days after the Friend agent splenic enlargement is apparently reduced but the evidence for this is based only on the results depicted in Table I.

The experiments cited, together with others, showed that when the inoculation into weanling Albany mice of Friend agent precedes that of MHV1 by several days the pathogenicity of MHV1 is much increased. In the following experiment this enhancement of pathogenicity was used as an indirect means of titration of the Friend agent and the result compared with the direct method. The dilutions of Friend agent were inoculated into groups of 20 mice and each group was split into equal parts to give two sets of titrations with an uninoculated control group for each set. Eight days later all mice of one set were inoculated with the usual dose of MHV1. The hepatitis in those which died and in the survivors killed on the sixth day was scored as described under Methods. The mice of the duplicate set were also killed 14 days after inoculation of Friend agent and, as indicated earlier, each mouse whose spleen was heavier than 0·2 g. was considered to be positive. The results are shown in Table II. The $ID_{50}$ by the indirect method was $10^{-4\cdot2}$ g. of spleen and by the direct method $10^{-5\cdot5}$ g. of spleen.

*Experiments with Moloney agent and MHV1*

Preliminary experiments with Moloney agent showed that it enhanced the virulence of MHV1 provided it was inoculated into baby mice 20–28 days before the MHV1 inoculation. In one experiment a group of 10 baby mice was inoculated with suspension of infected Moloney lymph node (M1/L). One died during lactation. The rest were weaned at 21 days old and given the usual inoculation of

TABLE II.—*Direct and Indirect Titration of Friend Agent*

| Dilution of Friend agent ($-\log_{10}$) | Indirect titration: Hepatitis scored, No. mice graded as 0 | 1 | 2 | 3 | 4 | 5 | Mortality $\frac{\text{Mice which died}}{\text{Mice in group}}$ | Infection rate $\frac{\text{Mice infected}}{\text{Mice in group}}$ | Direct titration: Infection rate $\frac{\text{Mice infected}}{\text{Mice in group}}$ |
|---|---|---|---|---|---|---|---|---|---|
| ∞ | 6 | 4 | 0 | 0 | 0 | 0 | 0/10 | 0/10 | 0/7 |
| 9 | 5 | 5 | 0 | 0 | 0 | 0 | 0/10 | 0/10 | 0/10 |
| 8 | 6 | 1 | 3 | 0 | 0 | 0 | 0/10 | 0/10 | 0/10 |
| 7 | 3 | 4 | 2 | 1 | 0 | 0 | 0/10 | 1/10 | 0/10 |
| 6 | 4 | 4 | 2 | 0 | 0 | 0 | 0/10 | 0/10 | 0/10 |
| 5 | 4 | 5 | 0 | 1 | 0 | 0 | 0/10 | 1/10 | 6/10 |
| 4 | 2 | 3 | 3 | 1 | 1 | 0 | 0/10 | 2/10 | 6/10 |
| 3 | 0 | 1 | 2 | 1 | 4 | 2 | 2/10 | 7/10 | 10/10 |
| 2 | 0 | 0 | 0 | 1 | 4 | 5 | 5/10 | 10/10 | 9/9 |
| 1 | 0 | 0 | 0 | 0 | 1 | 9 | 9/10 | 10/10 | 10/10 |
| $ID_{50}$ | | | | | | | | $10^{-4.2}$ g. | $10^{-5.5}$ g. |

*Indirect titration*

Usual dose of MHV1 inoculated 8 days after Friend agent.

Surviving mice sacrificed on 14th day after Friend inoculation and considered infected with Friend agent when hepatitis score $> 2$.

*Direct titration*

Killed on 14th day after Friend inoculation and considered infected with Friend agent when spleen weight $> 0 \cdot 2$ g.

MHV1. Six died of acute hepatitis on the 6th and 7th days and the livers of the remainder killed on the 7th day scored 3, 2 and 1. In another experiment, 8 baby mice were inoculated with Moloney suspension M1/S followed by MHV1 28 days later. When killed, all showed severe hepatitis and scored 3 or more.

An attempt was made to titrate Moloney agent by the potentiation of MHV1. Groups containing 10 or 11 baby mice were inoculated with dilutions of the Moloney lymph node preparation M2/L and a group of similar mice received an inoculation of SS broth only. Twenty-six days later, all mice were inoculated with the usual dose of MHV1 and after a further six days the degree of hepatitis scored in the usual way. The results, shown in Table III, led to a value of $10^{-6.4}$ g. of

TABLE III.—*Indirect Titration of Moloney Agent M2/L*

| Dilution of Moloney agent M2/L ($-\log_{10}$) | Hepatitis scored, No. mice graded as 0 | 1 | 2 | 3 | 4 | 5 | Infection rate $\frac{\text{Mice infected}}{\text{Mice in group}}$ |
|---|---|---|---|---|---|---|---|
| ∞ | 1 | 9 | 1 | 0 | 0 | 0 | 0/11 |
| 6 | 0 | 9 | 1 | 0 | 0 | 0 | 0/10 |
| 5 | 3 | 4 | 0 | 2 | 0 | 1 | 3/10 |
| 4 | 0 | 3 | 2 | 4 | 2 | 0 | 6/11 |
| 3 | 5 | 2 | 3 | 1 | 0 | 0 | 1/11 |
| 2 | 0 | 5 | 0 | 4 | 1 | 0 | 5/10 |
| 1 | 0 | 1 | 1 | 9 | 0 | 0 | 9/11 |
| $ID_{50}$ | | | | | | | $10^{-6.5}$ g. |

Usual dose of MHV1 inoculated 26 days after dilutions of Moloney agent.

Mice killed on 6th day after MHV1 inoculation and considered infected with Moloney agent when hepatitis score $> 2$.

tissue for the $ID_{50}$. In another indirect titration, groups of baby mice were inoculated with dilutions of Moloney suspension (M2/T). Twenty days later they were inoculated with MHV1 and the degree of hepatitis scored on the 6th day. The results, very similar to those of the last experiment, are shown briefly in Table IV

TABLE IV.—*Direct and Indirect Titration of Moloney Agent*

| Dilution of Moloney agent ($-\log_{10}$) | Indirect titration: Mice infected / Mice in group* | Direct titration: Mice infected / Mice in group* | Direct titration: Time of recognition of leukaemia (days from inoculation) | Direct titration: Mean (days) |
|---|---|---|---|---|
| ∞ | 0/7 | — | — | — |
| 7 | 0/9 | 0/9 | — | — |
| 6 | 1/9 | 1/7 | 157 | (157)† |
| 5 | 2/10 | 2/3 | 96, 157 | (127)† |
| 4 | 3/10 | 2/7 | 122, 146 | (134)† |
| 3 | 7/8 | 5/9 | 89, 122, 137, 143, 153 | 129 |
| 2 | 4/10 | 8/9 | 104, 122, 122, 122, 150, 153, 157, 157 | 136 |
| 1 | 7/10 | 7/8 | 94, 96, 104, 104, 122, 122, 157 | 114 |
| $ID_{50}$ g. | $10^{-4.6}$ | $10^{-4.9}$ | | |

*Indirect titration*

Albany baby mice (1–2 days) inoculated with Moloney suspension M2/T.

Usual dose of MHV1 inoculated 20 days after Moloney.

Mice killed on 6th day after MHV1 inoculation and considered infected with Moloney agent when hepatitis score >2.

*Direct titration*

Swiss baby mice (2–4 days) inoculated with Moloney suspension M2/T.

Mice with signs of leukaemia killed and diagnosis based on gross lesions. Remainder sacrificed 157 days after inoculation and examined for leukaemia.

* At the commencement all groups contained 10 baby mice. The majority of non-specific losses occurred during lactation.

† Means based on only two or less observations.

and led to an $ID_{50}$ of $10^{-4.6}$ g. of tissue. The experiment took 26 days. In order to obtain a direct titration, the dilutions of Moloney agent M2/T used in this experiment were also inoculated into groups of baby Swiss mice. As indicated in Methods, diagnosis of leukaemia was based upon lesions in mice killed during the currency of the experiment and in survivors sacrificed at its termination on the 157th day. The results, shown in detail in Table IV, led to an $ID_{50}$ of $10^{-4.9}$ g. of tissue.

### DISCUSSION

The enhancement of the pathogenicity of MHV1 by the infectious agent *E. coccoides* suggests that the enhancement produced by the tissue preparations containing Friend or Moloney leukaemia agents may be due to infectious agents. This view is supported by the fact that even dilute preparations containing Friend and Moloney agents enhance MHV1 and by the observation that Moloney agent must be inoculated as long as 20 or more days before MHV1 to increase its pathogenicity. Furthermore, the fact that for enhancement of MHV1 the times of pre-inoculation of preparations containing Friend agent and Moloney agent are considerably different suggests that different infectious agents are operative in each case and this indirectly supports the contention that Friend agent and

Moloney agent themselves are responsible for the enhancement. Nevertheless, the possibility that the enhancement is due to other factors present in the preparations cannot be excluded until purified preparations of Friend and Moloney agents are available.

*Eperythrozöon coccoides* enhances the pathogenicity of MHV1 during the period of high concentration of the parasite in mouse tissues (Gledhill, 1956). If this is also true for the enhancement of MHV1 by Friend and Moloney agents, it would imply that Friend agent attains a high titre in 4–7 days and Moloney agent in about 3 weeks. The point of interest, especially in the case of the Moloney agent, is that the virus would be present in high titre long before signs of leukaemia appear. For example, it might be that a certain cell-virus relationship of infrequent occurrence is required to produce the initial leukaemic cell. Indeed, if such were the case, most well adapted viruses which multiply over long periods of time with little or no direct pathogenic action might be potential agents of tumour production.

The capacity of Moloney agent to potentiate MHV1 is less marked than that of Friend agent or *E. coccoides*. Nevertheless it is sufficient to obtain a titration within a month and, since the time to produce leukaemia is so long and indefinite, this appears of considerable practical value. It would be interesting to determine whether other mouse tumour viruses potentiate MHV1.

The smaller amount of splenic enlargement when MHV1 is inoculated about 3–7 days before Friend agent could perhaps be ascribed to some form of viral interference. The smaller amount of splenic enlargement which probably occurs when MHV1 is inoculated 6 days after Friend agent is more difficult to explain ; it might be due to the capacity of MHV1 to parasitise and inhibit the division of tumour cells. Experiments to determine whether MHV1 interferes with Moloney agent in the same way as with Friend agent have not been undertaken. Unless the interference were much stronger than that of MHV1 towards Friend agent, it would be very difficult to detect since the onset of leukaemia occurs at times scattered over several months.

### SUMMARY

When Friend leukaemia agent was inoculated intraperitoneally into weanling mice from 4 to at least 8 days before intraperitoneal inoculation of mouse hepatitis virus (MHV1), the pathogenicity of the latter virus was much increased.

A similar but somewhat less marked enhancement of the pathogenicity of MHV1 occurred when 1–2 day old mice were inoculated with Moloney leukaemia agent 20–28 days before they were inoculated with MHV1.

The magnitude of potentiation of MHV1 with both Friend and Moloney agents depended on the strain of mice employed. In both cases it was greater in Albany than in Swiss mice.

The potentiation of MHV1 by these two agents can provide an indirect method of titration. With a suspension of Friend agent an $ID_{50}$ of $10^{-4.2}$ g. of spleen was obtained compared with $10^{-5.5}$ g. by a direct method based upon splenic enlargement. Two different suspensions of Moloney agent yielded $ID_{50}$s of $10^{-6.4}$ and $10^{-4.6}$ g. of tissue by the indirect method. The latter suspension titrated by the direct method gave an $ID_{50}$ of $10^{-4.9}$ g. and took 157 days compared with 26 days for the indirect titration.

MHV1 given 4–7 days *before* Friend agent reduced the amount of splenic enlargement which Friend agent produced in 14 days. MHV1 given 6 days *after* Friend agent also reduced the spleen weight but MHV1 given in the period from about 2 days before to 5 days after the Friend inoculation did not reduce the amount of splenic enlargement.

This work was supported by a grant from the National Foundation and Grant No. T-146 from the American Cancer Society. The author wishes to thank Dr. J. E. Hotchin for the invitation and provision of facilities to work in the virus department of the Division of Laboratories and Research, New York State Department of Health, Albany, N.Y.

The competent technical assistance of Miss Ruth Buckley is gratefully acknowledged.

### REFERENCES

Friend, Charlotte.—(1957) *J. exp. Med.*, **105**, 307.

Gledhill, A. W.—(1956) *J. gen. Microbiol.*, **15**, 292.

*Idem*, Dick, G. W. A. and Niven, Janet S. F.—(1955) *J. Path. Bact.*, **69**, 299.

Moloney, J. B.—(1960*a*) *J. nat. Cancer Inst.*, **24**, 933.—(1960*b*) Symposium : Phenomena of Tumor Viruses, U.S. Department of Health, Education & Welfare, p. 7.

Niven, Janet S. F., Gledhill, A. W., Dick, G. W. A. and Andrewes, C. H.—(1952) *Lancet*, ii, 1061.

Reed, L. J. and Muench, H.—(1938) *Amer. J. Hyg.*, **27**, 493.